# Impact of hypoxia on the double-strand break repair after photon and carbon ion irradiation of radioresistant HNSCC cells

**DOI:** 10.1038/s41598-020-78354-7

**Published:** 2020-12-07

**Authors:** Anne-Sophie Wozny, Gersende Alphonse, Audrey Cassard, Céline Malésys, Safa Louati, Michael Beuve, Philippe Lalle, Dominique Ardail, Tetsuo Nakajima, Claire Rodriguez-Lafrasse

**Affiliations:** 1 UMR CNRS5822/IN2P3, IP2I, PRISME, Laboratoire de Radiobiologie Cellulaire et Moléculaire, Faculté de Médecine Lyon-Sud, Univ Lyon, Université Lyon, 69921 Oullins Cedex, France; 2 Service de Biochimie et Biologie Moléculaire, Hospices Civils de Lyon, Centre Hospitalier Lyon-Sud, 69495 Pierre-Bénite, France; 3 Département de Radiothérapie, Institut de Cancérologie de la Loire Lucien Neuwirth, 42270 St Priest-en-Jarez, France; 4 UMR CNRS5822 /IN2P3, IP2I, PRISME, Univ Lyon, Université Lyon 1, 69322 Villeurbanne, France; 5 Department of Radiation Effects Research, National Institute of Radiological Sciences, National Institute for Quantum and Radiological Science and Technology, Inage-ku, Chiba, 263-8555 Japan

**Keywords:** Cancer microenvironment, Cancer stem cells, Head and neck cancer

## Abstract

DNA double-strand breaks (DSBs) induced by photon irradiation are the most deleterious damage for cancer cells and their efficient repair may contribute to radioresistance, particularly in hypoxic conditions. Carbon ions (C-ions) act independently of the oxygen concentration and trigger complex- and clustered-DSBs difficult to repair. Understanding the interrelation between hypoxia, radiation-type, and DNA-repair is therefore essential for overcoming radioresistance. The DSBs signaling and the contribution of the canonical non-homologous end-joining (NHEJ-c) and homologous-recombination (HR) repair pathways were assessed by immunostaining in two cancer-stem-cell (CSCs) and non-CSCs HNSCC cell lines. Detection and signaling of DSBs were lower in response to C-ions than photons. Hypoxia increased the decay-rate of the detected DSBs (γH2AX) in CSCs after photons and the initiation of DSB repair signaling (P-ATM) in CSCs and non-CSCs after both radiations, but not the choice of DSB repair pathway (53BP1). Additionally, hypoxia increased the NHEJ-c (DNA-PK) and the HR pathway (RAD51) activation only after photons. Furthermore, the involvement of the HR seemed to be higher in CSCs after photons and in non-CSCs after C-ions. Taken together, our results show that C-ions may overcome the radioresistance of HNSCC associated with DNA repair, particularly in CSCs, and independently of a hypoxic microenvironment.

## Introduction

Radiation induces cell death mainly through the induction of DNA damage. The capacity of cancer cells to repair these damage modulates the radioresistance of numerous solid tumors^1–3^. Among all types of DNA damage, DNA double-strand breaks (DSBs) are the most complex and deleterious for cells^4,5^. In fact, by affecting both DNA strands, the complementary undamaged strand that is needed as a template to restore the sequence in the damaged strand is unavailable, thus compromising the genome stability and leading to cell death, mutations, chromosome fusions, and tumorigenesis^1^. In response to radiation exposure, DSBs are first signaled by the phosphorylations of histone H2AX at Ser139 (γH2AX) and of the ataxia-telangiectasia mutated protein (ATM) at Ser1981 around the lesion sites^6,7^. This then allows the activation of the two major repair pathways of DSBs: the canonical non-homologous end-joining (NHEJ-c) and the homologous recombination (HR) pathways^8,9^. Although the choice of the pathway that is used for DSB repair is mainly regulated by the cell-cycle phase, other factors, such as chromatin structure, DNA end complexity, and transcriptional status around DSB sites, are also probably determinants^10^.

NHEJ repairs DSBs throughout the cell cycle, whereas HR is only active during the S/G2 phases following DNA replication, which partly explains why the NHEJ-c pathway is involved in the repair of ~ 70% of DSBs in human cells, whereas HR is used in the residual ~ 30%^9^. The error-prone NHEJ-c pathway involves the heterodimer Ku70/80, which instantly recognizes the DSB ends with high affinity, inhibits the initiation of HR, and recruits proteins such as DNA-PK and Artemis. Subsequently, the XRCC4-DNA ligase IV complex ligates the two DNA ends. It also activates the P53-binding protein 1 (53BP1), which dictates the DSB repair pathway by promoting NHEJ-mediated DSB repair while inhibiting HR^11,12^. The HR pathway after DSB signaling is mediated by the Mre11/Rad50/NSB1 (MRE) complex and end resection by the CtBP-interaction protein, followed by the stabilization of DNA strands by the RPA protein, which is then replaced by RAD51^8^.

During the conventional therapy with photons, the sparse ionization patterns caused by the low-linear energy transfer (LET) secondary electrons induce various types of DNA damage^13,14^. However, high-LET radiation types such as C-ions deposit their energy into the tracks of the particle, thus inducing more complex and clustered damage (CDS) compared with photons; consequently, they are more deleterious at biological levels (e.g., cell death, mutations, and cell-cycle arrest)^15–17^. Though CDS are generated by photons, their increasing frequency as well as their spatial distribution contribute to the superior biological efficacy of C-ions^18–21^. Numerous studies performed over the last decades, including ours, have highlighted the improved abilities of C-ions to kill cancer cells compared with photons, which may be attributed to an inefficient Ku-dependent NHEJ-c repair pathway^13,20,22,23^: C-ions might induce more damage in nearby DNA structures compared with photons, leading to short DNA DSB fragments (< 40 bp), which may prevent the efficient binding of Ku to the two ends of DNA, consequently delaying the NHEJ-c pathway while increasing the contribution of the HR pathway^17^.

Head and Neck Squamous Cell Carcinoma (HNSCC) is the sixth most common cancer worldwide. HNSCC is characterized by a low survival rate (below 30%) at 3 years^24^. Despite major advances in therapeutic strategies, intrinsic resistance and often late diagnosis at stages III–IV lead to local recurrences, relapses, and metastasis^25^. The presence of cancer stem cells (CSCs) in solid tumors, which preferentially activate the DNA damage response, leading to more efficient DNA repair, mainly explain these relapses; moreover, their location in hypoxic niches also contributes strongly to their radio- and chemoresistance^23,26–28^. Ionizing radiations produce various radicals that cause DSBs. Oxygen, the molecule with the highest affinity in cells, reacts extremely rapidly with these free radicals. Moreover, hypoxia sustains a “quiescent” state of the CSCs, thus protecting them from radiotherapy^5,10,29^. Our team showed previously that hypoxia increases radioresistance in non-CSCs and CSCs of HNSCC cell lines after photon exposure, which resulted in an oxygen enhancement ratio (OER) > 1, an increase in Reactive Oxygen Species (ROS) production, and the stabilization of the main regulator of the response to hypoxia, i.e., the HIF-1α protein^20^. In addition, our work and that of other researchers underlined the independent oxygen effect of C-ions, which resulted in an OER of ~ 1, particularly in HNSCC^20,30,31^. Several studies showed that hypoxia without irradiation increases the activities of ATM and DNA-PK through the Src and AMPK signaling pathways, independently of DSBs, thus suggesting an alternative mode of stimulation of ATM under hypoxic conditions^29,32–34^. The histone γH2AX has also been identified as a crucial player in hypoxia-driven neovascularization^35^, whereas other studies have shown that hypoxia associated to X-Rays induces the downregulation of HR and promotes NHEJ^29,36^.

The dynamics of the repair of low- and high-LET radiation-induced DSBs remains however poorly understood. The respective contributions of the NHEJ-c and HR pathways to the C-ion- and photon-induced DSBs have not been clarified. By studying the link between irradiation, hypoxia, and DNA repair, after C-ion and photon exposure, in two HNSCC cell lines and their CSCs subpopulations, our work may help to understand the differential hypoxia-related cell survival. Therefore, it will improve our knowledge about the radioresistance of HNSCC.

## Results

### Hypoxia increases the survival fraction in response to X-rays but not after C-ion exposure

**Figure 1:**
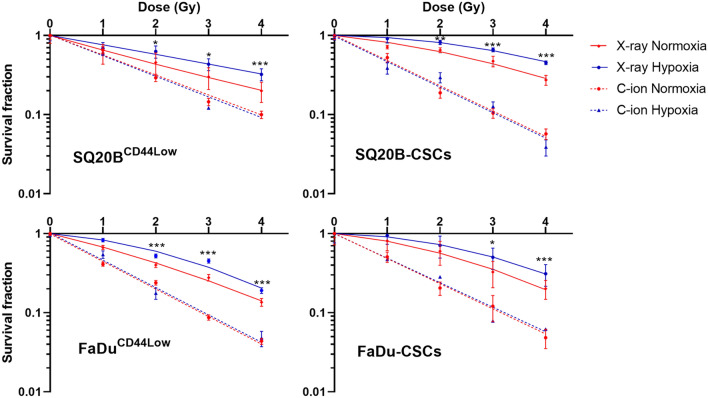
Clonogenic survival curves. SQ20B^CD44Low^, FaDu^CD44Low^, SQ20B-CSCs and FaDu-CSCs were exposed to photons or carbon ion irradiation under normoxia or chronic hypoxia. The survival fractions ± SD were presented in response to an irradiation of 1, 2, 3 and 4 Gy. Student’s *t*-test was performed between survival fractions of normoxia and hypoxia curves for each cell line (*P < 0.05, **P < 0.01 and ***P < 0.001).

Cell survival curves were established for the SQ20B^CD44Low^ and FaDu^CD44Low^ populations as well as for FaDu-CSCs and SQ20B-CSCs after photon and C-ion irradiation in normoxic and chronic hypoxic conditions (Fig. 1). The *D*_10_ value (dose required to kill 90% of the cell population), the RBE (relative biological effectiveness) and the oxygen enhancement ratio (OER) at 10% survival were presented in Table 1. A higher SF2 (survival fraction at 2 Gy) was observed in CSCs compared to non-CSCs, confirming their higher resistance to photons. As expected, after photon irradiation, the four HNSCC cell lines were significantly more resistant under hypoxia compared to normoxia (OER > 1). The better efficacy of carbon ions compared to photons was also confirmed by lower SF2, as well as a lower dependence on oxygen concentration (OER = 1).

**Table 1:** Radiobiological parameters.

	X-rays			Carbon ions			RBE
	SF2	D_10_	OER	SF2	D_10_	OER	
FaDu^CD44Low^ Normoxia	0.42	4.50	1.11	0.20	2.85	1.02	1.58
FaDu^CD44Low^ Hypoxia	0.59	4.98		0.21	2.91		1.71
FaDu-CSCs Normoxia	0.57	4.93	1.15	0.23	3.16	1.02	1.56
FaDu-CSCs Hypoxia	0.71	5.66		0.24	3.22		1.76
SQ20B^CD44Low^ Normoxia	0.44	6.00	1.32	0.32	3.97	0.97	1.51
SQ20B^CD44Low^ Hypoxia	0.58	7.90		0.31	3.86		2.05
SQ20B-CSCs Normoxia	0.62	5.90	1.19	0.23	3.13	0.99	1.88
SQ20B-CSCs Hypoxia	0.82	7.05		0.22	3.09		2.28

Survival Fractions at 2 Gy (SF2) and D10 values were calculated according the linear-quadratic model in response to photon or carbon ion irradiation under normoxia or chronic hypoxia in the four HNSCC subpopulation studied. The RBE and the OER were also calculated at 10% of cell survival.

### Hypoxia increases the initiation of the DSBs repair signaling in response to X-ray and C-ion exposure

**Figure 2:**
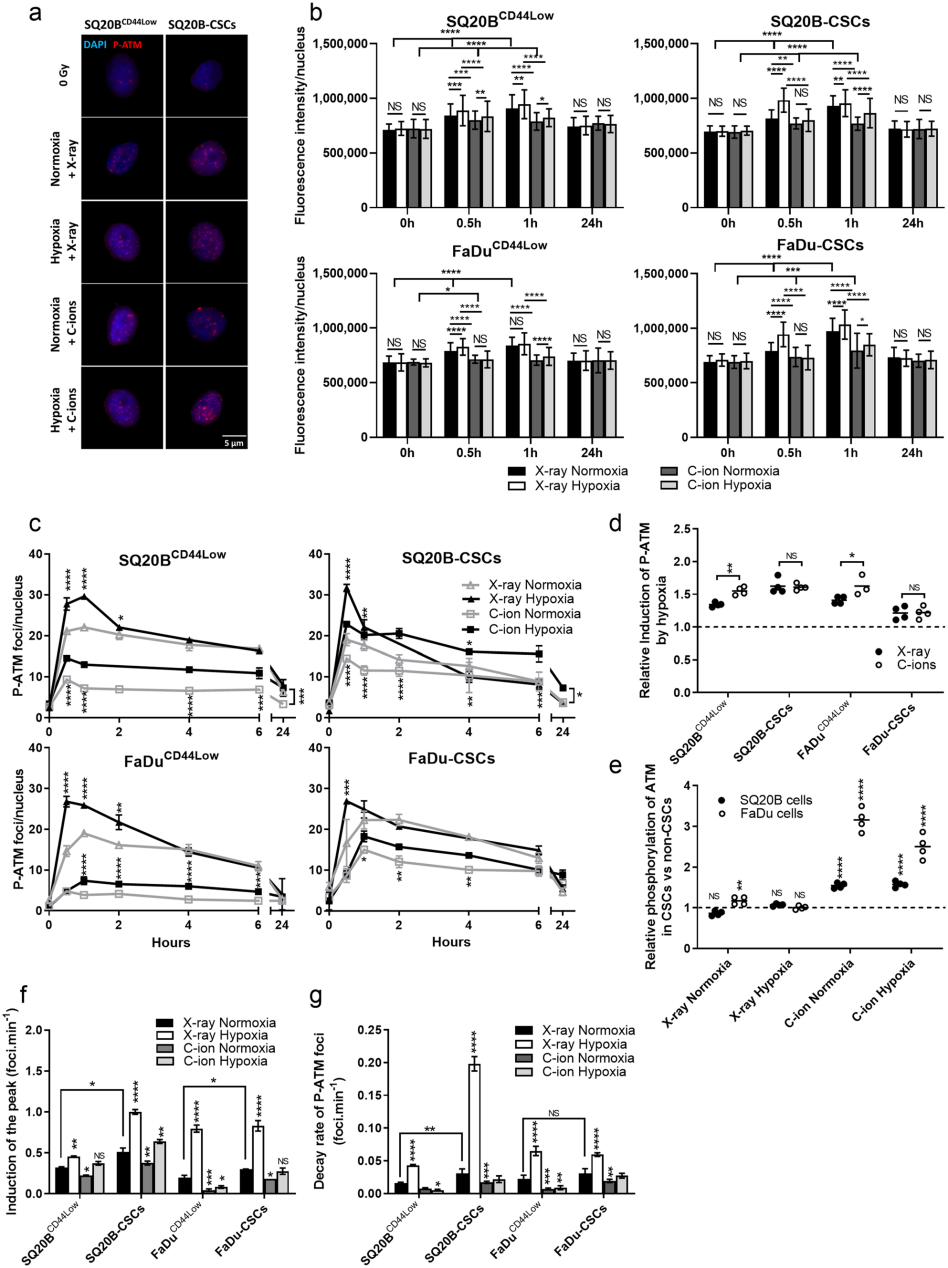
Hypoxia increases the initiation of DSBs repair signaling in response to X-ray and C-ion exposure. (**a**) Representative images of the detection of DSBs characterized by P-ATM foci at peak after X-ray or C-ion irradiation in normoxic and hypoxic conditions in SQ20B^CD44Low^ and SQ20B-CSCs cells (Metafer 4 v3.13.4). (**b**) P-ATM fluorescence intensities. The means of the fluorescence intensities between different oxygenation conditions were compared for both types of irradiation, as well as the activation of the phosphorylation by irradiation with the 0 h condition (two-way ANOVA test). (**c**) Kinetics of P-ATM foci induction and decay from 30 min to 24 h after X-ray and C-ion irradiation in normoxic and hypoxic conditions. The symbols indicate means and the error bars indicate SD values. The means of the different oxygenation conditions were compared for both types of irradiation (two-tailed Student’s t-test). (**d**) Relative induction of the P-ATM foci peak in response to hypoxia vs. normoxia for each condition of irradiation, as determined from the data shown in (**c**) (two-way ANOVA test). (**e**) Relative induction of the P-ATM foci peak in CSCs compared to relative non-CSCs for each condition of irradiation and oxygenation, as determined from the data shown in (**c**) (two-way ANOVA test). (**f**) Induction speed of the P-ATM foci from basal levels to peak, as determined from the data shown in (**c**). The means and error bars (SD values) are presented and each condition was statistically compared with the X-ray normoxic condition, as well as the non-CSC and CSC conditions under normoxia in the presence of X-rays (two-way ANOVA test). (**g**) Decay rate of the P-ATM foci from the peak level to half of the peak value, as determined from the data shown in (**c**). The means and error bars (SD values) are presented and each condition was statistically compared to the X-ray normoxic condition (two-way ANOVA test). *P < 0.05, **P < 0.01, ***P < 0.001, ****P < 0.0001 (n = 2).

To investigate the effects of hypoxia on the signaling of the DDR (DNA Damage Response), the phosphorylation of the ATM protein (P-ATM) was studied in SQ20B^CD44Low^, FaDu^CD44Low^, SQ20B-CSCs, and FaDu-CSCs after exposure to 2 Gy X-rays or C-ions under normoxia or hypoxia (Fig. 2). Representative images of the immunostaining of P-ATM at 30 min are presented in Fig. 2a for SQ20B^CD44Low^and SQ20B-CSCs, and the means of the fluorescence intensities of P-ATM per nucleus at 30 min, 1 h and 24 h are presented in Fig. 2b. The higher fluorescence intensities observed with photons indicated a more important phosphorylation of ATM compared with C-ions at peak. The P-ATM intensities were also increased at peak under hypoxia after both irradiations. The kinetics of foci from 30 min to 24 h after both types of irradiation are shown for the four populations in Fig. 2c. The values of each hypoxic time point (mean ± SD) were statistically compared to the relative normoxic mean. Confirming the results obtained with fluorescence intensities, the P-ATM levels of foci were systematically higher in response to photons compared with C-ions, and hypoxia triggered a higher level of phosphorylation of ATM, suggesting a better DSBs repair signaling in response to both types of irradiation in normoxic condition. As an example, in SQ20B^CD44Low^, the peak under normoxia detected 1 h after photon exposure (22.05 ± 0.53) reached a value of 29.60 ± 0.55 under hypoxia, whereas this peak (30 min) was earlier and decreased to 9.35 ± 0.13 (normoxia) and 14.51 ± 0.66 (hypoxia) in response to C-ions. In SQ20B-CSCs, the values for the early peak (30 min) were of 19.05 ± 1.47 (normoxia X-rays) and 31.67 ± 0.87 (hypoxia X-rays) and decreased to 14.44 ± 0.74 under normoxia and 22.82 ± 0.73 under hypoxia after C-ion exposure. The results depicted in Fig. 2d confirmed the hypothesis that hypoxia enhances the P-ATM levels, regardless of cell line and type of radiation. The induction of the P-ATM peak, localized between 30 min and 1 h depending on the cell population, was not significantly different between X-rays and C-ions under hypoxia; however, the difference was more marked in non-CSCs after C-ion than photon exposure, thus suggesting that non-CSCs, which are not located in a hypoxic microenvironment, are more sensitive to hypoxia. Regarding the relative P-ATM levels associated with stemness (CSC value peak compared with relative non-CSC peak in the same condition of irradiation and oxygen concentration), Fig. 2e shows that P-ATM was increased in CSCs compared with non-CSCs in response to C-ions in both oxygenation conditions, whereas no significant differences were observed after photon exposure. Figure 2f shows that the induction of the peak of P-ATM was faster in CSCs compared with non-CSCs and that hypoxia triggered a faster detection of DSBs in all the populations in response to photons. This rate was decreased in normoxic and hypoxic conditions for all populations after C-ion exposure. Finally, we investigated the decay rate of P-ATM foci from the peak to half-value of the peak for each experimental condition (Fig. 2g). Hypoxia triggered a more prominent decay rate of the phosphorylation of ATM compared with normoxia in response to X-ray exposure in the four populations, whereas C-ions decreased significantly this decay rate in normoxic and hypoxic conditions. Taken together, our results show that hypoxia increases the initiation of DSBs repair signaling, particularly after photon exposure. Moreover, CSCs are able to exert DSBs repair signaling more importantly compared with non-CSCs after C-ion exposure.

### Hypoxia increases the decay of γH2AX foci only in CSCs after X-ray but not C-ion exposure

**Figure 3:**
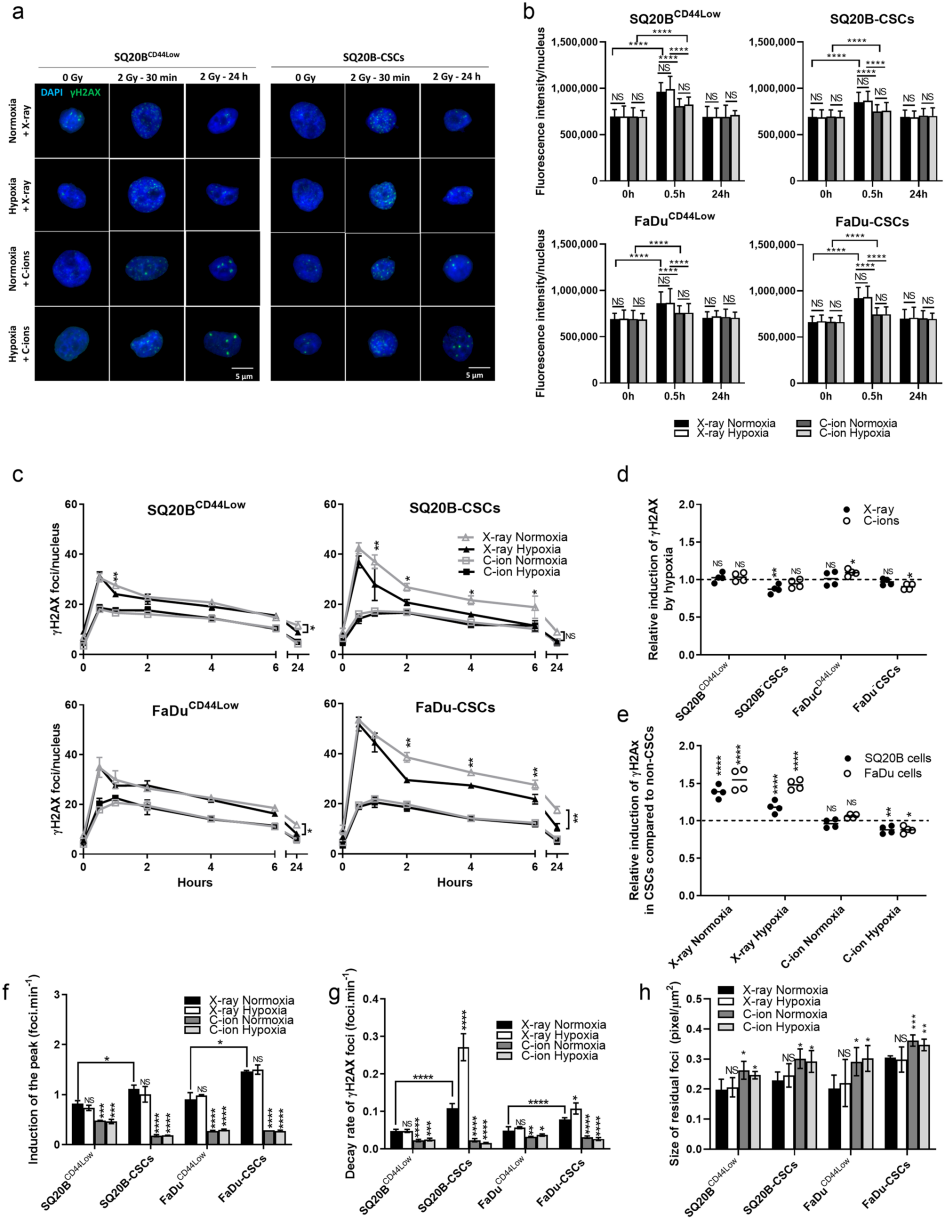
Hypoxia increases the decay-rate of γH2AX foci in CSCs after photons and not after C-ions. (**a**) Representative images of the DSBs characterized by γH2AX foci at peak and residual foci at 24 h after X-ray or C-ion irradiation in normoxic and hypoxic conditions for SQ20B^CD44Low^ and SQ20B-CSCs cells (Metafer 4 v3.13.4). (**b**) γH2AX fluorescence intensities. The means of the fluorescence intensities between different oxygenation conditions were compared for both types of irradiation, as well as the activation of the phosphorylation by irradiation with the 0 h condition (two-way ANOVA test). (**c**) Kinetics of γH2AX foci induction and decay from 30 min to 24 h after X-ray and C-ion irradiation in normoxic and hypoxic conditions. The symbols indicate means and the error bars indicate SD values. For the same type of irradiation, the means of the normoxic and hypoxic conditions were compared between themselves (two-tailed Student’s *t*-test). (**d**) Relative induction of the γH2AX peak under hypoxia compared with normoxia for each condition of irradiation, as determined from the data shown in (**c**) (two-way ANOVA test). (**e**) Relative induction of the γH2AX peak in CSCs compared with relative non-CSCs for each condition of irradiation and oxygenation, as determined from the data shown in (**c**) (two-way ANOVA test). (**f**) Induction rate to reach the peak of γH2AX in all conditions. The means and error bars (SD values) are presented and each condition was statistically compared to the X-ray normoxic condition, as well as the non-CSC and CSC conditions in normoxic conditions with X-rays (two-way ANOVA test). (**g**) Decay rate of the γH2AX foci from the peak level to half of the peak value, as determined from the data shown in (**c**). The means and error bars (SD values) are presented and each condition was statistically compared to the X-ray normoxic condition (two-way ANOVA test). (**h**) Size of the residual foci. The means and error bars (SD values) are presented and each condition was statistically compared with the X-ray normoxic condition (two-tailed Student’s *t*-test). *P < 0.05, **P < 0.01, ***P < 0.001, ****P < 0.0001 (n = 3 for photons and n = 2 for C-ions).

The detection of DSBs after radiation exposure was studied in normoxic and hypoxic conditions through the expression of γH2AX (Fig. 3). Representative images of the peak of induction (30 min) and the residual γH2AX foci (24 h) obtained by immunostaining are presented for SQ20B^CD44Low^ and SQ20-CSCs in Fig. 3a. The means of the fluorescence intensities per nucleus of γH2AX are shown in Fig. 3b. These fluorescence intensities at peak were higher in response to photons compared with C-ions without any modulation by hypoxia for the 4 cell populations studied. Then, γH2AX kinetics were assessed in both oxygenation conditions from 30 min to 24 h after 2 Gy X-ray or C-ion exposure in SQ20B^CD44Low^, FaDu^CD44Low^, and their respective CSC subpopulations (Fig. 3c). For all populations studied and regardless of time point (30 min to 24 h), a lower amount of γH2AX foci was counted in response to C-ions compared with photon exposure. For example, in normoxic cells, 30.63 ± 1.67 and 42.42 ± 2.14 foci were counted at 30 min in SQ20B^CD44Low^ and SQ20B-CSCs in response to 2 Gy X-rays vs. 18.00 ± 0.17 and 17.29 ± 1.23 foci in response to C-ions, respectively. For all the populations and regardless of experimental condition, the initial peak of γH2AX foci was obtained 30 min after radiation exposure. However, if the proportion of the initial peak was not or only slightly affected by hypoxia, the stemness status seemed to modulate it (Fig. 3d,e). In fact, the relative induction of the γH2AX peak, calculated between non-CSCs and CSCs in response to both types of irradiation in normoxic and hypoxic conditions, showed that photons induced a greater number of foci in CSCs compared with non-CSCs in both the normoxic and hypoxic conditions. Interestingly, this differential response between CSCs and non-CSCs was abrogated in response to C-ions in both oxygenation conditions, supporting the enhanced biological efficiency of C-ions for killing CSCs. Moreover, Fig. 3f shows the induction speed of the initial peak established for all experimental conditions. Under normoxia, CSCs reached the maximum γH2AX level faster than did non-CSCs in response to X-rays (0.83 ± 0.06 foci min^−1^ for SQ20B^CD44Low^, 1.12 ± 0.07 foci min^−1^ for SQ20B-CSCs, 0.91 ± 0.13 foci min^−1^ for FaDu^CD44Low^, and 1.47 ± 0.02 foci min^−1^ for FaDu-CSCs). Hypoxia did not modify the results after exposure to photons, whereas the speed of induction of the peak after C-ions exposure was significantly reduced for all the populations in normoxic and hypoxic conditions (Fig. 3f), corresponding to a lower rate of detection of the DSBs (Fig. 3c). From 30 min to 6 h, although hypoxia combined with X-ray exposure compared with normoxia did not modulate the number of γH2AX foci in non-CSCs, this proportion decreased in SQ20B-CSCs and FaDu-CSCs under hypoxia (Fig. 3c). In response to C-ions, the kinetics of γH2AX were not modulated by hypoxia, which was in accordance with their lower dependence on oxygen concentration. Figure 3g shows that the γH2AX decay rate from the early peak to half value was increased in CSCs compared with non-CSCs in response to X-ray exposure and that hypoxia combined with X-rays compared with normoxia increased the γH2AX decay rate in CSCs. This was not observed in non-CSCs. In response to C-ions, the decay rates were significantly decreased under normoxia and hypoxia in the four cellular populations. Additionally, when residual γH2AX foci were significantly decreased by hypoxia versus normoxia for all the populations after X-ray exposure (Fig. 3c), it seemed that the residual γH2AX foci were not affected by hypoxia in response to C-ion exposure. Finally, the size of the residual foci was quantified (Fig. 3h) and showed bigger foci in response to C-ions compared with photons, without impact of the oxygen concentration. Altogether, our results show that the detection of the DSBs through γH2AX is independent of the tension in oxygen after C-ions, but the decay of γH2AX foci is increased in CSCs after X-rays in hypoxic conditions.

### The expression of 53BP1, a key regulator of the DSB repair pathway, is not modulated by hypoxia

**Figure 4:**
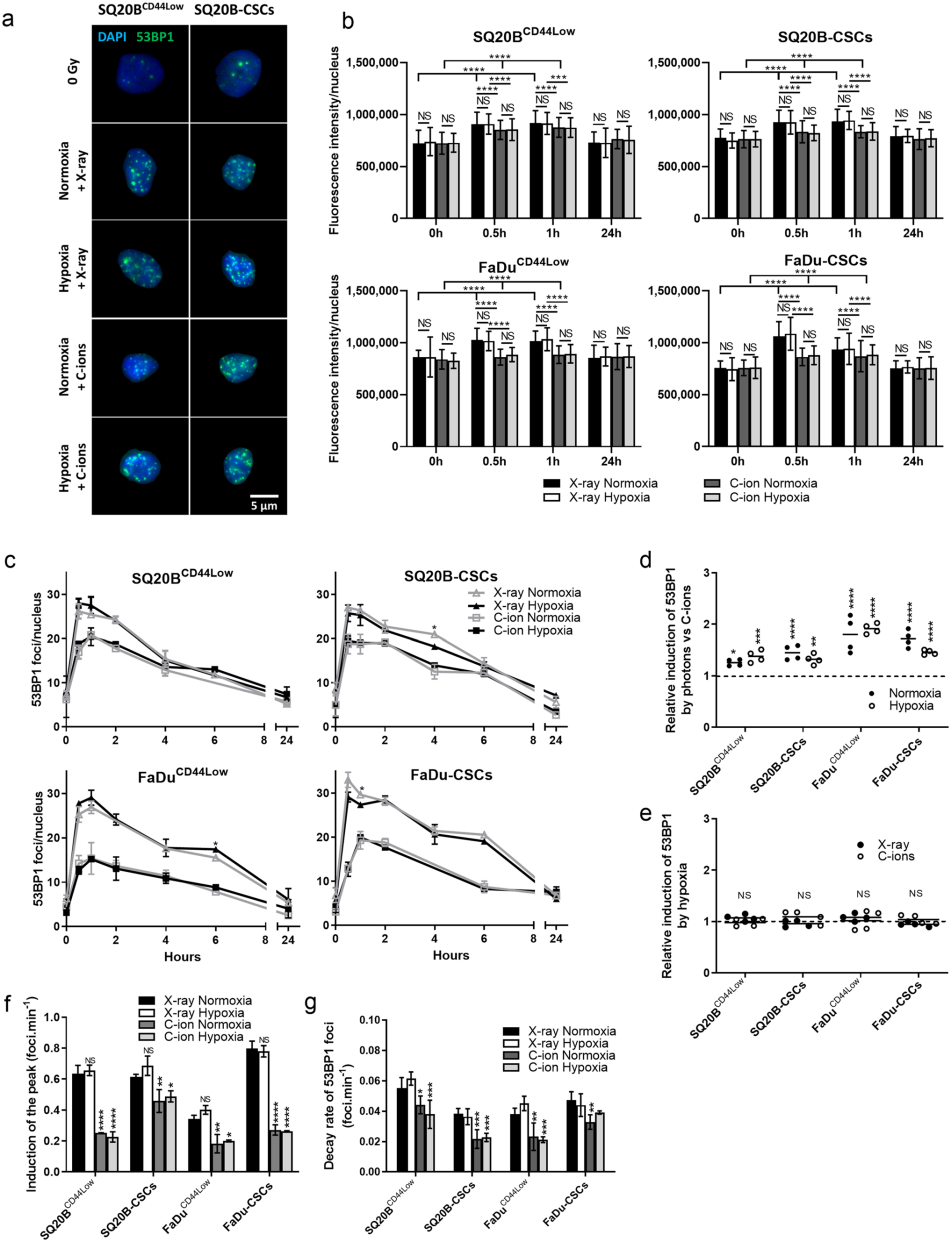
C-ions induce 53BP1 foci to a lesser extent than do photons, for both independently of oxygen concentration.(**a**) Representative images of 53BP1 foci at their peaks after 2 Gy X-ray or C-ion irradiation in normoxic and hypoxic conditions for SQ20B^CD44Low^ and SQ20B-CSCs cells (Metafer 4 v3.13.4). (**b**) 53BP1fluorescence intensities. The means of the fluorescence intensities between different oxygenation conditions were compared for both types of irradiation, as well as the induction of 53BP1 by irradiation with the 0 h condition (two-way ANOVA test). (**c**) Kinetics of 53BP1 foci induction and decay from 30 min to 24 h after X-ray and C-ion irradiation in normoxic and hypoxic conditions. The symbols indicate means and the error bars indicate SD values. The means of the different oxygenation conditions were compared for both types of irradiation (two-tailed Student’s t-test). (**d**) Relative induction of 53BP1 foci peak in response to photons compared with C-ions for each condition of irradiation and oxygenation, as determined from the data shown in (**c**) (two-way ANOVA test). (**e**) Relative induction of the 53BP1 foci peak in response to hypoxia for each condition of irradiation, as determined from the data shown in (**c**) (two-way ANOVA test). (**f**) Rate of the induction of the 53BP1 peak from the basal levels, as determined from the data shown in (**c**). The means and error bars (SD values) are presented and each condition was statistically compared to the X-ray normoxic condition (two-way ANOVA test). (**g**) Decay rate of the 53BP1 foci from the peak level to half of the peak value, as determined from the data shown in (**b**). The means and error bars (SD values) are presented and each condition was statistically compared to the X-ray normoxic condition (two-way ANOVA test). *P < 0.05, **P < 0.01, ***P < 0.001, ****P < 0.0001 (n = 2).

After investigating the role of hypoxia on the observed foci of DSBs, we focused on its role in the DNA repair pathways choice via 53BP1 expression (Fig. 4). Representative images of the immunostaining of 53BP1 foci detected 30 min after radiation exposure are presented in Fig. 4a for SQ20B^CD44Low^ and SQ20B-CSCs, whereas the means of the fluorescence intensities per nucleus are shown in Fig. 4b. The fluorescence intensities indicated a higher expression of 53BP1 in response to photons compared with C-ions without any modulation by hypoxia. The kinetics from 30 min to 24 h after exposure to both types of irradiation are shown for the four populations in Fig. 4c. In accordance with the results obtained for γH2AX and P-ATM, as well as the fluorescence intensities, this analysis showed that the number of 53BP1 foci was greater in response to photons compared to C-ions, regardless of the oxygen concentration. In response to photons under normoxia, the peaks of 53BP1 foci at 30 min were 26.18 ± 1.64 in SQ20B^CD44Low^ and 27.03 ± 0.48 in SQ20B-CSCs, whereas they were delayed at 1 h and decreased to 20.85 ± 0.143 and 19.19 ± 0.67, respectively, in response to C-ions under normoxia. Figure 4d confirms the better activation of 53BP1 in response to photons compared with C-ions in the four populations. Figure 4e shows that there was no significant modification of the activation of 53BP1 in hypoxic conditions compared with normoxia for all the populations and both types of irradiation. Moreover, Fig. 4f reveals that hypoxia did not modulate the rate of induction of the peak in response to photons, whereas C-ions significantly decreased it, regardless of the oxygenation condition. Similarly, hypoxia did not affect the decay rate of 53BP1 foci, whereas C-ions decreased it significantly (Fig. 4g). These results suggest that hypoxia does not affect the triggering of the DNA repair pathway through 53BP1.

### Consequences of photon and C-ion irradiation on cell-cycle phases under normoxia and hypoxia

**Figure 5:**
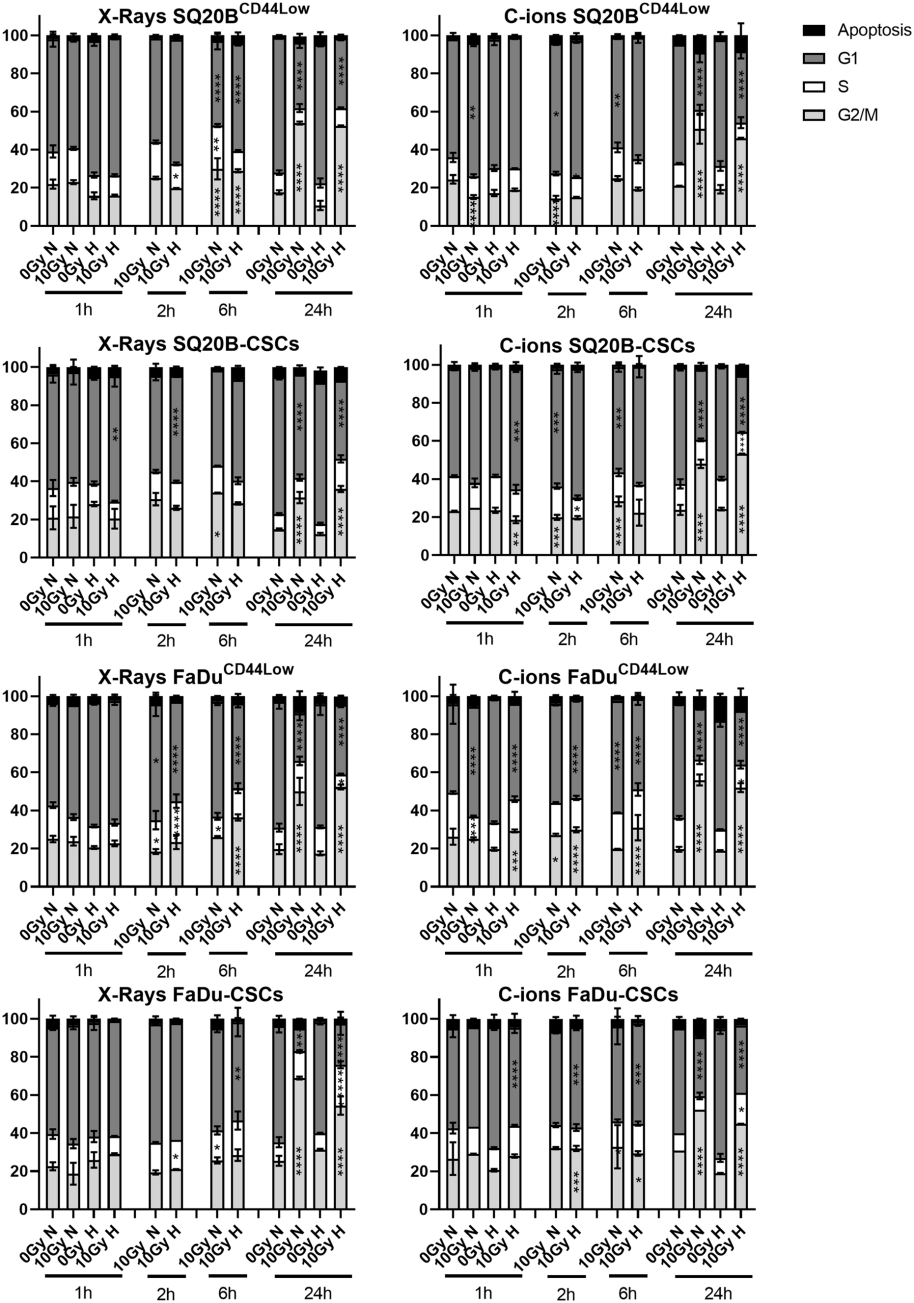
Repartition of the HNSCC cells in the different phases of the cell cycle. Cell-cycle analyses were performed for SQ20B^CD44Low^, SQ20B-CSCs FaDu^CD44Low^ and FaDu-CSCs, in response to photons and C-ions in normoxic (N) and hypoxic conditions (H). Cells were grown under normoxia or hypoxia, irradiated with 10 Gy, and prepared for analysis by flow cytometry at 1, 2, 6, and 24 h after irradiation. The means and error bars (SD values) are presented and each condition was statistically compared to their respective non-irradiated condition (two-way ANOVA test). *P < 0.05, **P < 0.01, ***P < 0.001, ****P < 0.0001 (n = 3).

The four cell populations were irradiated with photons or C-ions under normoxic or hypoxic conditions and the percentage of cells in each phase of the cell cycle was quantified 1 h to 24 h post-irradiation using a flow cytometer (Fig. 5, Supplementary Tables S1 and S2). Our results show that the majority of the cells were in the G1 phase during the experiments from 1 to 6 h after exposure, with a very low proportion of cells being in the sub-G1 phase, mainly at 24 h post-irradiation. Moreover, hypoxia did not significantly modulate the cell-cycle arrest, whereas an increased arrest in the G2/M phase occurred in all populations 24 h after exposure to both types of irradiation, regardless of the oxygenation condition.

### Hypoxia increases the recruitment of the DNA-PK protein (NHEJ-c pathway) in response to photons, but not after exposure to C-ions regardless of the oxygen concentration

**Figure 6:**
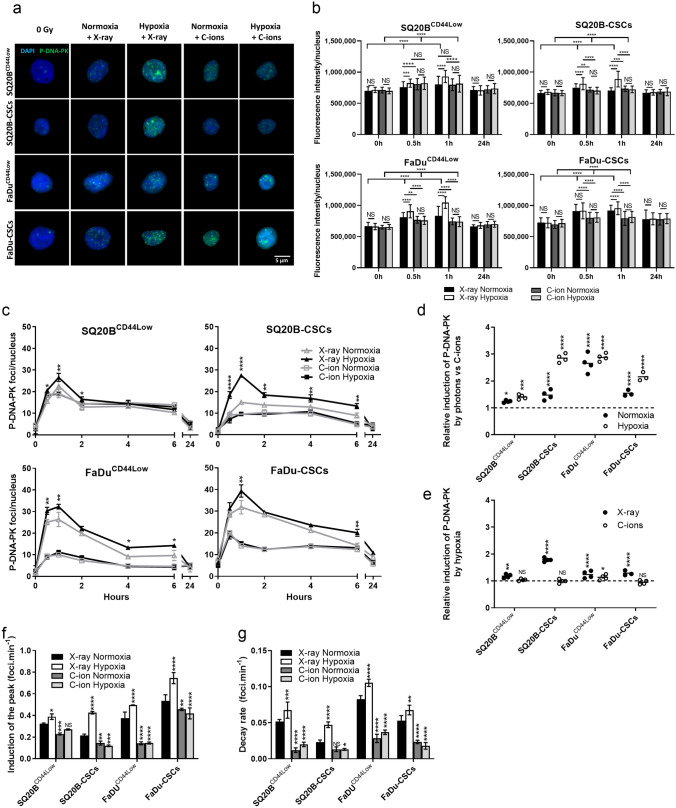
Hypoxia triggers the canonical non-homologous end-joining pathway through P-DNA-PK with photons and not with C-ions. (**a**) Representative images of P-DNA-PK foci at the peak after X-ray or C-ion irradiation in normoxic and hypoxic conditions for the four subpopulations studied (Metafer 4 v3.13.4). (**b**) P-DNA-PK fluorescence intensities. The means of the fluorescence intensities between different oxygenation conditions were compared for both types of irradiation, as well as the phosphorylation of DNA-PK by irradiation with the 0 h condition (two-way ANOVA test). **(c)** Kinetics of P-DNA-PK foci induction and decay from 30 min to 24 h after X-ray and C-ion irradiation in normoxic and hypoxic conditions. The symbols indicate means and the error bars indicate SD values. The means of the different oxygenation conditions were compared for both types of irradiation (two-tailed Student’s t-test). (**d**) Relative induction of the P-DNA-PK foci peak in response to photons compared with C-ions for each condition of irradiation and oxygenation, as determined from the data shown in (**c**) (two-way ANOVA test). (**e**) Relative induction of the P-DNA-PK foci peak in response to hypoxia for each condition of irradiation, as determined from the data shown in (**c**) (two-way ANOVA test). (**f**) Rate of the induction of the P-DNA-PK peak from the basal levels, as determined from the data shown in (**c**). The means and error bars (SD values) are presented and each condition was statistically compared to the X-ray normoxic condition (two-way ANOVA test). (**g**) Decay rate of the P-DNA-PK expression from the peak to half of the peak value, as determined from the data shown in (**c**). The means and error bars (SD values) are presented and each condition was statistically compared to the X-ray normoxic condition (two-way ANOVA test). *P < 0.05, **P < 0.01, ***P < 0.001, ****P < 0.0001 (n = 2).

The effect of hypoxia on the activation of the NHEJ-c pathway was studied, based on the phosphorylation of DNA-PK (P-DNA-PK) (Fig. 6). Figure 6a shows a representative image of the immunostaining of P-DNA-PK in SQ20B^CD44Low^, FaDu^CD44Low^, and their respective populations of CSCs detected 1 h after a 2 Gy exposure to X-rays or C-ions in normoxic or hypoxic conditions. The means of the fluorescence intensities per nucleus and the kinetics study performed from 30 min to 24 h after irradiation in the four cell populations are respectively shown in Fig. 6b,c. The intensities and curves obtained show that photons increased the phosphorylation of DNA-PK to a greater extent than did C-ions and that hypoxia enhanced it only in response to photon exposure (Fig. 6b,c). Regarding the relative induction of P-DNA-PK, Fig. 6d shows that the P-DNA-PK peak was more prominent in response to photons compared with C-ions in the four cellular populations. Moreover, Fig. 6e confirmed the hypothesis that hypoxia triggers a greater activation of DNA-PK in response to photons compared with C-ions, regardless of the cell population. The quantification of both the mean rate of reaching the peak from basal levels (Fig. 6f) and the decay rate from the peak to the half-value of the peak (Fig. 6g) showed that hypoxia enhanced significantly both the induction and the decay rates in the four cell populations in response to photons, thus suggesting that hypoxia contributes to the recruitment of the NHEJ-c pathway and to superior efficiency. However, both the induction and the decay rates of the peak were significantly decreased in response to C-ions in most of the oxygenation conditions, thus confirming the hypothesis that the biological efficacy of C-ions may partially depend on the decrease of the activation of the NHEJ-c pathway.

Taken together, these results show that hypoxia enhances the NHEJ-c pathway by promoting a faster phosphorylation of DNA-PK after photon exposure, whereas the response to C-ions is independent of the oxygen concentration.

### Hypoxia increases the recruitment of RAD51 (HR pathway) in response to photons, but not to C-ions

**Figure 7:**
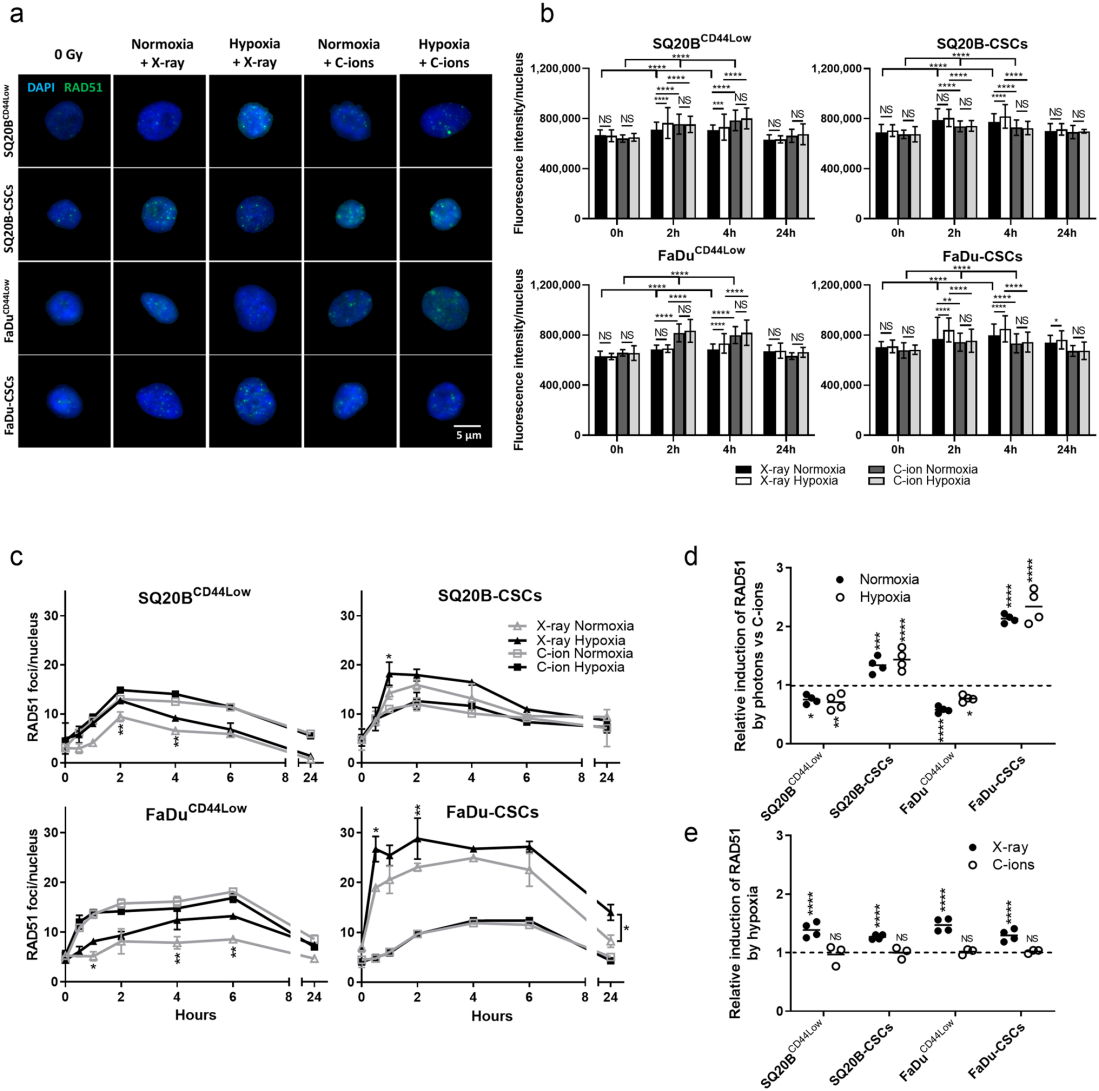
Hypoxia enhances the homologous recombination pathway after photons whereas C-ions act independently of the oxygen concentration. (**a**) Representative images of RAD51 foci at the peak after X-ray or C-ion irradiation in normoxic and hypoxic conditions for the four subpopulations studied (Metafer 4 v3.13.4). (**b**) RAD51 fluorescence intensities. The means of the fluorescence intensities between different oxygenation conditions were compared for both types of irradiation, as well as the induction of RAD51 by irradiation with the 0 h condition (two-way ANOVA test). (**c**) Kinetics of RAD51 foci induction and decay from 30 min to 24 h after X-ray and C-ion irradiation in normoxic and hypoxic conditions. The symbols indicate means and the error bars indicate SD values. The means of the different oxygenation conditions were compared for both irradiations (two-tailed Student’s t-test).(**d**) Relative induction of the RAD51 foci peak in response to photons compared with C-ions for each condition of irradiation and oxygenation, as determined from the data shown in (**c**) (two-way ANOVA test). (**e**) Relative induction of the RAD51 foci peak in response to hypoxia for each condition of irradiation, as determined from the data shown in (**c**) (two-way ANOVA test). *P < 0.05, **P < 0.01, ***P < 0.001, ****P < 0.0001 (n = 2).

As RAD51 is an essential effector of the HR pathway, we then investigated its expression from 30 min to 24 h after exposure to both types of irradiation under normoxic and hypoxic conditions in the two CSCs and their respective non-CSCs populations (Fig. 7). As shown for the other markers, representative images of the immunostaining of RAD51 are presented (Fig. 7a) for SQ20B^CD44Low^, SQ20B-CSCs, FaDu^CD44Low^, and FaDu-CSCs, while the mean of the fluorescence intensities per nucleus are shown in Fig. 7b for 0 h, 2 h, 4 h and 24 h. The kinetic studies performed from 30 min to 24 h after exposure to both types of irradiation are summarized in Fig. 7b. First, these graphs highlight the presence of some spreading of the RAD51 peak between 1 and 6 h after exposure, which was consistent with the later activation of the HR compared with the NHEJ pathway. They also show the more pronounced activation of RAD51 in hypoxic conditions after photon exposure in all populations, whereas hypoxia did not modify these profiles in response to C-ions. These observations are supported by Fig. 7b. We then calculated the relative induction of RAD51 foci in response to both types of irradiation in the four subpopulations in normoxic and hypoxic conditions (Fig. 7d). After exposure to photons, the ratios were > 1 for SQ20B-CSCs and FaDu-CSCs both in normoxic and hypoxic conditions, thus confirming the higher activation of the HR pathway in these two populations compared to C-ions. In non-CSCs, the ratios were < 1, which was in accordance with the lower RAD51 foci detected in response to photons compared with C-ions and higher activation of the HR with C-ions.

Figure 7e shows that the ratio of hypoxic/normoxic values of the peak of RAD51 foci was > 1 in response to photons in the four cell populations, whereas hypoxia did not affect this ratio in response to C-ions. Moreover, according to Fig. 7b, it seems that the foci induction of RAD51 was increased in response to C-ions in non-CSCs, whereas its expression was upregulated in response to photons in CSCs.

These data suggest that hypoxia triggers the activation of the HR pathway in response to photons, whereas C-ions induce HR independently of the oxygen concentration. Furthermore, the involvement of the HR pathway in the repair of DSBs seems to be different between CSCs and non-CSCs according to the type of radiation used.

## Discussion

In solid tumors, cancer cells and particularly CSCs acquire genetic and adaptive changes that lead to their survival and proliferation in a hypoxic microenvironment^34^. Numerous studies have established that hypoxia is notably associated with a poor prognosis in HNSCC, mainly because of an increase in chemo- and radioresistance in hypoxic cells^36^. Hypoxia has been proposed to be a direct inducer of DNA damage by repressing genes that are essential for DNA repair, such as the downregulation of the DNA mismatch repair gene *MLH1*^37^ or the functional decrease of the nucleotide excision repair pathway^38^. C-ion irradiation constitutes an alternative to conventional radiotherapy, as the biological effects of C-ions would be less dependent on the oxygen concentration, according to the LET and the type of cancer^31^. Previous works performed by our team have confirmed the superior biological efficacy of C-ions on HNSCC-CSCs and non-CSCs compared with photons (increased cell death, decreased invasion–migration processes, etc.)^20,21,23,39^. It was also evidenced that hypoxia, associated with HIF-1α expression, increases cell survival in response to photon exposure, whereas this latter is less dependent on the oxygen concentration after C-ion exposure^20^. Given these results, investigating the detection, signaling, and the choice of the NHEJ or HR repair pathway after low- and high-LET exposure in non-CSCs and CSCs seemed essential to understand the mechanisms that lead to radioresistance in HNSCC and therefore to develop better-targeted radiosensitizing therapies.

First, the exertion of DSB repair signaling was assessed through the phosphorylation of ATM in response to photons and C-ions under normoxic and hypoxic conditions. Our results showed that hypoxia increased the phosphorylation of ATM to a greater extent compared with normoxia, regardless of the type of radiation. Several studies have demonstrated that hypoxia increases P-ATM expression^32,35,40,41^ independently of the MRN complex^33,41^, which suggests an alternative mode of stimulation in the absence of DSBs. Moreover, as hypoxia contributed to the acceleration of the formation and decay of P-ATM foci in response to photons, C-ion irradiation decreased significantly these rates in both oxygenation conditions. These findings are in agreement with the recent report by Maalouf *et al.*^14^, who concluded that LET and the radiation/particle type affect the formation of the ATM monomers in the cytoplasm, that are required for DSB recognition. Our results also show that the signaling of DSBs was faster in CSCs compared with non-CSCs in response to photons under normoxia, thus confirming their superior DNA repair abilities.

Next, we focused on the detection of the DSBs via γH2AX expression, which led us to quantify an increase in DSBs in CSCs compared with non-CSCs in response to photons, regardless of the oxygenation condition. This differential response between CSCs and non-CSCs was abrogated after exposure to C-ions, thus confirming the superior biological efficacy of C-ions on CSCs compared with photons. Moreover, the DSBs observed by γH2AX were detected and disappeared more rapidly in CSCs after photon exposure, which suggests a better activation of the downstream DNA repair pathways, regardless of the oxygenation condition. In response to C-ions, the values at the peak and the decay of γH2AX expression were significantly decreased in both CSCs and non-CSCs. Numerous studies reported that DNA damage is more complex in response to high LET^42,43^. In fact, as highlighted by Lopez Perez *et al.*^44^, who quantified γH2AX by flow-cytometry analysis, conventional fluorescence microscopy, and super-resolution microscopy, mainly this latter is able to detect DSBs in clusters compared with basic fluorescence microscopes. However, in this study, we used a fluorescence microscope that is able to generate a Z-stack, which reduced the risk of underestimating the number of foci observed in 2D. Moreover, previous experiments enabled us to correlate the number of foci with FACS analyses. Like Gerelchuluun *et al.*, we observed no differences in residual foci at 24 h between photon and C-ion irradiation at physical equivalent doses^45^, which was in contrast with the study performed by Suetens *et al.* using human prostate adenocarcinoma cells, in which the number of residual foci remained elevated 24 h after C-ion irradiation^19^. The induction of DSBs, as represented through Monte-Carlo simulations in response to high LET, showed that the number of DSB clusters increased as their complexity according to LET^17^. Using super-resolution microscopy, another study found that, in response to high LET, the structural organization of the DSBs included sub-foci that were associated with the induction of densely-spaced DSBs, leading to a better biological efficacy of C-ions^46^. These data explain the lower proportion of γH2AX foci detected after C-ions which certainly corresponds to clusters of damage, supported by the quantification of bigger foci in response to C-ions compared with photons, this independently of the oxygen concentration. Additionally, the fluorescence intensities per nucleus presented for all the markers studied at different time points confirmed the higher activation of the signaling pathways in response to photons compared with C-ions.

After the detection and signaling of the DSBs, 53BP1 is usually considered as a mediator of DSB signaling that promotes NHEJ-c repair and inhibits HR by antagonizing BRCA1. The choice of the DSB repair pathway is a complex process that depends on repair protein proficiencies, cell-cycle phase, and cell-cycle checkpoint control^47^. In this study, we revealed that photons activated 53BP1 foci induction expression to a greater extent than did C-ions, independently of the oxygen concentration. Moreover, C-ions seemed to induce a decrease in the formation and disappearance of 53BP1 foci, suggesting a lower activation of the NHEJ-c repair pathway and a lower inhibition of the HR pathway in the presence of high LET. The acetylation of histones and the post-modification of 53BP1 were recently reported as a mechanism of regulation of 53BP1 accumulation at DNA break ends^12^. Furthermore, the localization of 53BP1 and the size of RAD51 foci are not significantly different after low- and high-LET irradiation^48^. Other studies have addressed the different contributions of the NHEJ-c and HR pathways to DSB repair according to the cell-cycle phase in which cells are irradiated and the complexity of the DSBs^45,49,50^. In our work, the cell-cycle phase was also monitored, and the results showed that cells were mainly in the G1 phase during our experiments, with an increased G2/M arrest observed 24 h after exposure to both types of irradiation. Moreover, hypoxia did not appear to modulate the cell-cycle phase in the four populations. In vitro studies have shown a more pronounced cell-cycle arrest induced by high-LET particles compared with photons^51,52^. While cells are more prone to the induction of DSBs by photons during the G2/M phase, the response of cancer cells to C-ions does not depend on the cell-cycle phase^19,51,53^.

In the present study, the NHEJ-c pathway was assessed through the phosphorylation of DNA-PK. Compared with C-ions, photons enhanced P-DNA-PK expression to a greater extent (pic maximum and peak-rate), and this effect was further enhanced under hypoxia. Bouquet *et al.* have proposed a model in which the activation of DNA-PK under hypoxia mainly induces changes in chromatin structure, initiating DNA-PK activation in the absence of DNA lesions. However, they cannot exclude the possibility that DNA DSBs partly contribute to DNA-PK activation via a synergic mechanism^29,33^. Several studies have also clarified that Ku70/80 binding protects the DNA ends from unnecessary resection and inhibits HR initiation^45^, although the HR pathway appeared to be involved to a greater extent than that expected. Our experiments showed that photons are stronger triggers of the HR pathway by RAD51 compared with C-ions, with a hypoxia-induced overactivation. However, in the two non-CSC populations, C-ions induced RAD51 expression to a greater extent than did photons, which suggests the existence of a differential mechanism between CSCs and non-CSCs. In the present case, CSCs may activate the NHEJ-c and HR pathways in response to photons, especially under hypoxia. According to our results, the non-CSCs would be more dependent on the NHEJ-c pathway; thus, the recruitment of RAD51 in the HR pathway would be decreased. C-ions lead to more fragmented DNA lesions, so that the involvement of HR in the repair of these lesions would be greater than that observed for the lesions caused by photons. However, the involvement of other DNA repair pathways such as NHEJ-a (alternative) or SSA (single-strand annealing), especially in response to C-ions, should be further investigated. As reported previously, this HR activation occurs later than that of the NHEJ-c pathway^54^.

The oxygen concentration is also a main parameter involved in the contribution of each pathway to DSB repair. Cells under severe hypoxia, which could be qualified as anoxia, show different responses compared with those observed under moderate hypoxia (0.5–2.0%)^29,34^. Conflicting results have been reported after moderate hypoxia, depending on the cancer cell model, the duration and timing of the hypoxia, and the oxygen concentration, with both the HR and NHEJ-c pathways being either strongly activated or inhibited^29^.

Several previous studies performed by our team enabled us to propose the stealth-bomber paradigm to explain the specific effects of carbon ions. It relies on the ROS distribution at the nanometric scale immediately after irradiation, which is dense and homogenous in response to photons, and condensed in clusters around the tracks in response to carbon ions^20,21,39,55^. We presented evidence of the bomber effect by the absence of an oxygen effect^20^ and a dose-rate effect after C-ion exposure^56,^ and the presence of increased cell death in HNSCC cells and their stem-cell subpopulation^20,23,26^. It also supports the fact that the telomeric status did not impact the sensitivity of glioblastoma cells to C-ions contrary to photons. At the opposite, the stealth effect results from the absence of ROS outside the tracks, which should explain the absence of activation of the invasion/migration pathways or those of cell survival^20,21^ and the lower detection and signaling of DSBs demonstrated in the present work.

In summary, hypoxia improves the signaling of DSBs (P-ATM) in non-CSCs and CSCs after exposure to both types of radiation, whereas DSBs detection through γH2AX increases only in CSCs after photon exposure. Moreover, the choice of repair pathway (53BP1) is not modulated by hypoxia, regardless of the type of radiation. Finally, hypoxia increases the triggering of the NHEJ-c (DNA-PK) pathway in both populations, but also the HR (RAD51) only in CSCs in response to photon exposure. In response to C-ions, the DSB repair mechanism is less dependent on the oxygen concentration regarding the signaling, the choice, and the involvement of NHEJ-c or HR repair pathways. Moreover, the HR pathway seems to be less involved in non-CSCs after C-ions exposure compared with that observed after photon exposure. Taken together, our results show that C-ions may help overcome the radioresistance of HNSCC cells associated with DNA repair, particularly in CSCs, and independently of the hypoxic microenvironment.

## Methods

### Cell culture

The SQ20B and FaDu radioresistant cell lines, which were established from HNSCC tumors, were provided by Prof. John Little (Boston, MA, USA) or purchased from the American Type Culture Collection (ATCC, USA), respectively. The CSC subpopulations SQ20B-CSCs, and FaDu-CSCs were obtained by cell sorting^20,57^. The stem-like characteristics of CSCs were validated in vitro by the formation of tumorspheres^23,57^, the expression of genes such as *β-catenin* or *Bmi1*^23,57^, the resistance to chemo- and or radiotherapies^20,23,56,58^, the invasion/migration abilities^21,39^, and their capacity to form tumors after injection in mice^23,59^. Compared with SQ20B-CSCs, the parental SQ20B cell line, which contains < 1% of CD44-positive cells, was chosen as the non-CSC control and is termed SQ20B^CD44Low^ hereafter. As the FaDu parental cell line contains more than 20% CD44-positive cells^59^, the subpopulation of FaDu CD44-negative cells, termed FaDu^CD44Low^, was obtained after cell sorting and considered as the non-CSC control. All cells were cultured as previously described^20,57^.

### *TP53* and *CDKN2A* genotyping

As the *TP53* gene plays a major role in determining radiosensitivity, the mutational status of our cell lines was also analyzed at the platform of molecular biology of Lyon-Sud Hospital (France). For FaDu^CD44Low^ and FaDu-CSCs, the mutations reported by ATCC were found in the two populations (*CDKN2A*, c.151-1G>T homozygous; *TP53*, c.376-1G>A heterozygous; c.743G>T heterozygous). The *TP53* c.743G>T mutation was also found at the heterozygous status in SQ20B non-CSCs and CSCs.

### Irradiation

Cells were irradiated with X-rays at Lyon-Sud Medical School (France) using an X-RAD320 irradiator (250 kV)^57^. Cell culture flasks and labteks (Falcon, USA) were horizontally irradiated with 2 Gy at a dose rate of 2 Gy min^−1^. Carbon ion beam time was granted by the National Institute of Radiological Sciences of Chiba (NIRS, Japan) using their heavy ion medical accelerator (HIMAC; Chiba, Japan)^60^. Irradiation of cells was performed vertically at 2 Gy min^−1^ using a carbon ion beam energy of 290 MeV/n, center of 6 cm Spread-out Bragg Peak (SOBP) with average LET 50 keV µm^−1^. Time points were realized from 30 min after irradiation (time necessary to eliminate neutrons in the irradiation room) to 24 h. As RBEs were dependent on the subpopulations tested and the conditions of oxygenation (data supported by a previous study using C-ions of 75 MeV/n and the survival curves performed at NIRS at an energy of 290 MeV/n in Fig. 1), all experiments with C-ions were performed at NIRS at the equivalent physical dose that was used for X-ray irradiation, i.e., 2 Gy^20^. Institutional safety was approved and followed for radiation and culture of human cell lines.

### Hypoxic conditions

Cells were maintained at 37 °C with 5% CO_2_, which constitutes normoxic conditions. Hypoxic conditions were obtained in a tri-gas chamber (Heracell 150i, Thermo Fisher Scientific, Waltham, MA, USA; or 9000E; Wakenyaku, Kyoto, Japan) under an atmosphere containing 1% O_2,_ 5% CO_2_, and 94% N_2_. Cells were cultured in normoxic conditions or hypoxia (incubation for 20–24 h before irradiation and from 30 min to 24 h for kinetic studies after irradiation). During irradiation, to limit oxygen exchange, labteks were covered with a plastic film and non-filtered caps were used, and then opened before being  replaced in the incubator.

### Colony formation assays

Cell survival curves were assessed by the standard colony formation assay as previously described^20^. After irradiation, cells were seeded in 6-well plates or flasks of 25 cm^2^. Colonies containing at least 64 cells after 6 cellular divisions were counted with a Colcount system (Optronix, Oxford, UK). Survival curves were calculated according to the formula of the linear-quadratic model *S* = exp (− *αD* − *βD*^2^), where *S* is the survival fraction and *D* the dose expressed in Gray. For C-ion irradiation, the curves were fitted with *S* = exp^(−*αD*)^. The SF2 and the D_10_ were determined thanks to these curves. The RBE (ratio of the D_10_ in response to photons and C-ions) and the OER (ratio of the *D*_10_ values between chronic hypoxia and normoxia) were then calculated.

### Immunofluorescence

The day before experiments, cells were grown on labteks at a density of 3 × 10^5^ cells per slide and incubated in normoxic or hypoxic conditions. From 30 min to 24 h after 0 Gy or 2 Gy irradiation in normoxic or hypoxic conditions, cells were washed twice with PBS, then fixed for 15 min in 4% paraformaldehyde (Santa Cruz Biotechnologies, Dallas, TX, USA). The chamber slides were washed three times with PBS and permeabilized in 0.2% triton X-100/PBS for 5 min for RAD51, 53BP1, γH2AX, and phosphorylated DNA-PK (P-DNA-PK); or in 0.4% triton X-100/PBS for 15 min for phosphorylated ATM (P-ATM). After washing three times with PBS/0.1% Tween 20/0.05% triton X-100 or PBS alone for P-ATM, the non-specific sites were blocked after incubation for 10 min in a buffer containing PBS/2% milk/fetal bovine serum (FBS)/0.1% triton X-100 for RAD51 and γH2AX; PBS/0.2% milk/FBS-0.1% triton X-100 for 53BP1; and for 1 h in PBS/1% bovine serum albumin for P-ATM. Slides were incubated with primary antibodies diluted in their respective blocking buffers for 1 h at room temperature with gentle agitation. The antibodies used for immunofluorescence were mouse anti-phospho-histone H2AX-Ser139 (1:1000; Merck, Kenilworth, NJ, USA), rabbit anti-RAD51 (1:750; Abcam, Cambridge, GB), rabbit anti-53BP1 (1:250; NovusBio, Littleton, CO, USA), rabbit anti-phospho-ATM-S1981 (1:250; Abcam), and rabbit anti-phospho-DNA-PKcs-S2056 (1:500; Abcam). Cells were rinsed three times for 5 min in the first washing solution and samples were incubated in secondary antibodies, anti-IgG rabbit Alexa Fluor 488 (1:500; Invitrogen, Carlsbad, CA, USA) or anti-IgG mouse Alexa Fluor 555 (1:250; Abcam), in blocking buffer for 1 h in the dark at room temperature. Cells were washed twice for 5 min in PBS/0.1% Tween 20 or three times for 5 min in PBS for P-ATM. Subsequently, cells were incubated with 1 µg ml^−1^ of DAPI (Sigma, Kawasaki, JP) in PBS for 15 min and washed three times for 5 min in PBS. Cells were mounted using Fluoromount (Merck) and conserved at room temperature in the dark until analysis.

### Microscopy

Microscopy analyses were performed using the automated reading platform Metafer (MetaSystems GmbH, Altlußheim, DE), which comprises a motorized optical microscope (Axio Imager.Z2; Carl Zeiss, DE) with a monochromatic camera (CCD CoolCubeR 1 m; MetaSystems GmbH) and a 200 V dimmable mercury lamp (X-citeR; Excelitas Technologies, Waltham, MA, USA). For every condition, the fluorescent signals (foci) of at least 300 nuclei were quantified in duplicate and using at least two independent experiments. The signal quantified for P-ATM, RAD51, γH2Ax, P-DNA-PK, and 53BP1 represented the number of foci per nucleus. For each protein studied, the induction speed of the peak was determined from basal levels to the peak values and the decay-rate of the foci was calculated from the number of foci at peak to half of the peak value in foci min^−1^. The size of the residual γH2AX foci was quantified by Metafer Analysis and the global fluorescence intensities per nucleus were calculated using Image J software (NIH, USA).

### Cell cycle

Cells were grown in T25 flasks under normoxia or hypoxia and then irradiated with 10 Gy photons or C-ions. Cells were then incubated from 30 min to 24 h under both oxygenation conditions. The percentage of cells in each phase of the cell cycle was quantified using DAPI labeling. Adherent and floating cells were pooled, and after washing with PBS, they were fixed in 70% ice-cold ethanol for at least 24 h. Cells were then washed with PBS and resuspended in DAPI. Analyses were performed using an LSRII flow cytometer (BD Biosciences, San Jose, CA, USA) and at least 10,000 events were counted for each condition (n = 3) with a flow-rate of 200 events s^−1^.

### Statistical analysis

To analyze the results of the experiments, a two-way ANOVA multiple comparison test or a two-tailed Student’s *t*-test was performed to assess the significance of differences between two groups, with correction for multiple comparisons performed using the Holm–Sidak method. *P* values ≤ 0.05 were considered statistically significant (GraphPad Prism 8 and Excel Software). The levels of significance are indicated by asterisks, as follows: ****P < 0.0001, ***P < 0.001, **P < 0.01, and *P < 0.05.

## Supplementary information

Supplementary Tables.

## Data Availability

The datasets generated during and/or analysed during the current study are available from the corresponding author on reasonable request.
