# Supplementary material for: Impact of hypoxia on the double-strand break repair after photon and carbon ion irradiation of radioresistant HNSCC cells
Source: Sci Rep. 2020 Dec 7;10:21357. doi: 10.1038/s41598-020-78354-7 (PMC7721800; doi:10.1038/s41598-020-78354-7)
Supplement: Supplementary file 1 — Supplementary Tables. [file 41598_2020_78354_MOESM1_ESM.docx]

# Impact of hypoxia on the Double Strand Break repair after photon and carbon ion irradiation of radioresistant HNSCC cells

# Anne-Sophie Wozny^1, 2^, Gersende Alphonse^1, 2^, Audrey Cassard^1^, Céline Malesys^1^, Safa Louati^1^, Michael Beuve^3^, Philippe Lalle^1^, Dominique Ardail^1, 2^, Tetsuo Nakajima^4^ and Claire Rodriguez-Lafrasse^1, 2,^*

^1^Univ Lyon, Université Lyon, UMR CNRS5822/IN2P3, IP2I, PRISME, Laboratoire de Radiobiologie Cellulaire et Moléculaire, Faculté de Médecine Lyon-Sud, 69921 Oullins Cedex, France

^2^ Hospices Civils de Lyon, Service de Biochimie et Biologie Moléculaire, Centre Hospitalier Lyon-Sud, 69495 Pierre-Bénite, France

^3^Département de Radiothérapie, Institut de Cancérologie de la Loire Lucien Neuwirth, 42270 St Priest-en-Jarez, France

^4^Univ Lyon, Université Lyon 1, UMR CNRS5822 /IN2P3, IP2I, PRISME, PHABIO, 69322 Villeurbanne, France

^5^Department of Radiation Effects Research, National Institute of Radiological Sciences, National Institute for Quantum and Radiological Science and Technology, Inage-ku, Chiba 263-8555, Japan

^*^ claire.rodriguez-lafrasse@univ-lyon1.fr

**Supplementary Table S1.** Repartition of the HNSCC cells according to the cell-cycle phases. Cell-cycle analyses were performed for SQ20B^CD44Low^ and SQ20B-CSCs, in response to photons and C-ions in normoxic and hypoxic conditions. Cells were grown under normoxia or hypoxia, irradiated with 10 Gy, and prepared for analysis by flow cytometry at 1, 2, 6, and 24 h after irradiation (n = 3).

|  |  | SQ20B^CD44Low^ | | | | SQ20B-CSCs | | | |
| --- | --- | --- | --- | --- | --- | --- | --- | --- | --- |
|  |  | X-Rays | | C-ions | | X-Rays | | C-ions | |
|  |  | Normoxia | Hypoxia | Normoxia | Hypoxia | Normoxia | Hypoxia | Normoxia | Hypoxia |
| 0h 0Gy | G2/M: | 21.95 ± 2.35 | 15.81 ± 1.81 | 24.45 ± 2.23 | 17.25 ± 1.65 | 20.91 ± 6.04 | 28.17 ± 1.15 | 23.16 ± 0.26 | 23.58 ± 1.36 |
|  | S: | 17.12 ± 3.20 | 10.88 ± 1.36 | 11.48 ± 2.46 | 13.11 ± 1.55 | 15.62 ± 4.25 | 10.71 ± 0.14 | 18.33 ± 0.57 | 18.03 ± 0.65 |
|  | G1: | 58.91 ± 3.93 | 70.35 ± 2.62 | 61.91 ± 0.30 | 67.43 ± 2.92 | 59.54 ± 4.25 | 55.91 ± 1.53 | 56.43 ± 0.92 | 56.83 ± 0.97 |
|  | Apoptosis: | 2.02 ± 0.62 | 2.96 ± 0.66 | 2.16 ± 1.21 | 2.21 ± 0.67 | 3.93 ± 1.05 | 5.21 ± 0.14 | 2.08 ± 1.44 | 1.56 ± 0.48 |
| 1h 10Gy | G2/M: | 23.03 ± 1.04 | 15.93 ± 0.49 | 15.16 ± 0.99 | 18.97 ± 0.65 | 21.69 ± 6.04 | 20.44 ± 5.21 | 24.89 ± 0.00 | 18.63 ± 1.85 |
|  | S: | 17.68 ± 0.75 | 10.60 ± 0.63 | 11.03 ± 0.89 | 11.28 ± 0.05 | 18.08 ± 2.09 | 9.08 ± 0.41 | 13.09 ± 2.28 | 16.04 ± 2.31 |
|  | G1: | 57.16 ± 1.22 | 71.96 ± 0.68 | 69.70 ± 1.58 | 68.49 ± 0.68 | 57.56 ± 6.53 | 65.62 ± 5.42 | 60.28 ± 1.51 | 63.24 ± 1.68 |
|  | Apoptosis: | 2.13 ± 0.90 | 1.51 ± 0.54 | 4.11 ± 0.63 | 1.26 ± 0.23 | 2.67 ± 0.02 | 4.86 ± 0.61 | 1.74 ± 0.77 | 2.09 ± 1.30 |
| 2h 10Gy | G2/M: | 25.16 ± 0.75 | 19.75 ± 0.20 | 14.46 ± 1.39 | 14.97 ± 0.26 | 30.74 ± 3.25 | 26.18 ± 1.07 | 19.89 ± 1.34 | 19.72 ± 0.88 |
|  | S: | 18.84 ± 0.86 | 12.76 ± 0.79 | 13.04 ± 0.95 | 10.68 ± 0.32 | 14.38 ± 0.91 | 13.64 ± 0.61 | 16.39 ± 1.44 | 10.51 ± 1.19 |
|  | G1: | 54.39 ± 0.11 | 65.26 ± 0.54 | 68.12 ± 0.73 | 71.50 ± 1.10 | 50.26 ± 2.42 | 55.61 ± 0.67 | 61.42 ± 2.49 | 67.51 ± 1.63 |
|  | Apoptosis: | 1.61 ± 0.21 | 2.23 ± 0.28 | 4.38 ± 0.14 | 2.85 ± 1.16 | 4.62 ± 1.73 | 4.57 ± 0.21 | 2.30 ± 0.51 | 2.26 ± 1.19 |
| 6h 10Gy | G2/M: | 29.98 ± 5.57 | 28.92 ± 0.85 | 24.87 ± 1.27 | 19.33 ± 0.89 | 34.11 ± 0.03 | 28.46 ± 0.64 | 28.28 ± 2.54 | 22.36 ± 6.84 |
|  | S: | 22.67 ± 0.80 | 10.40 ± 0.49 | 16.35 ± 2.57 | 15.72 ± 2.17 | 14.08 ± 0.07 | 11.87 ± 1.75 | 15.18 ± 1.96 | 14.76 ± 0.96 |
|  | G1: | 44.36 ± 4.38 | 56.39 ± 1.03 | 57.55 ± 0.69 | 63.60 ± 2.61 | 50.01 ± 0.05 | 52.93 ± 0.53 | 55.28 ± 2.46 | 61.82 ± 5.57 |
|  | Apoptosis: | 2.99 ± 0.82 | 4.29 ± 1.49 | 1.23 ± 0.61 | 1.35 ± 0.43 | 1.8 ± 0.02 | 6.73 ± 0.63 | 1.25 ± 0.21 | 1.06 ± 0.31 |
| 24h 0Gy | G2/M: | 17.64 ± 1.13 | 10.80 ± 2.47 | 20.99 ± 0.12 | 19.26 ± 2.28 | 14.84 ± 0.54 | 12.41 ± 0.67 | 23.82 ± 2.65 | 24.25 ± 0.69 |
|  | S: | 10.37 ± 1.16 | 11.74 ± 2.47 | 11.91 ± 0.15 | 12.22 ± 2.60 | 8.03 ± 0.55 | 5.29 ± 0.65 | 13.68 ± 2.43 | 16.00 ± 0.95 |
|  | G1: | 70.87 ± 0.77 | 72.57 ± 0.96 | 62.23 ± 0.12 | 65.88 ± 0.40 | 71.75 ± 1.13 | 73.88 ± 0.44 | 61.08 ± 1.69 | 58.88 ± 1.07 |
|  | Apoptosis: | 1.12 ± 0.13 | 4.89 ± 1.54 | 4.87 ± 0.05 | 2.64 ± 1.69 | 5.38 ± 0.07 | 6.58 ± 1.59 | 1.42 ± 0.31 | 0.87 ± 0.21 |
| 24h 10Gy | G2/M: | 54.60 ± 0.79 | 52.47 ± 0.33 | 51.01 ± 7.87 | 45.94 ± 0.28 | 31.54 ± 2.99 | 36.04 ± 1.60 | 47.98 ± 2.22 | 53.28 ± 0.10 |
|  | S: | 7.81 ± 2.10 | 9.35 ± 0.42 | 9.93 ± 2.67 | 8.26 ± 2.88 | 10.51 ± 1.66 | 15.79 ± 1.93 | 12.56 ± 0.70 | 11.45 ± 0.03 |
|  | G1: | 34.33 ± 2.74 | 36.53 ± 0.90 | 30.06 ± 5.17 | 37.39 ± 3.69 | 53.95 ± 0.77 | 40.97 ± 0.34 | 36.88 ± 0.63 | 29.47 ± 0.01 |
|  | Apoptosis: | 3.26 ± 1.43 | 1.65 ± 0.33 | 9.00 ± 0.02 | 8.41 ± 6.30 | 4.07 ± 0.90 | 7.20 ± 0.01 | 2.58 ± 0.93 | 5.80 ± 0.02 |

**Supplementary Table S2.** Repartition of the HNSCC cells according to the cell-cycle phases. Cell-cycle analyses were performed for FaDu^CD44Low^ and FaDu-CSCs, in response to photons and C-ions in normoxic and hypoxic conditions. Cells were grown under normoxia or hypoxia, irradiated with 10 Gy, and prepared for analysis by flow cytometry at 1, 2, 6, and 24 h after irradiation (n = 3).

|  |  | FaDu^CD44Low^ | | | | FaDu-CSCs | | | |
| --- | --- | --- | --- | --- | --- | --- | --- | --- | --- |
|  |  | X-Rays | | C-ions | | X-Rays | | C-ions | |
|  |  | Normoxia | Hypoxia | Normoxia | Hypoxia | Normoxia | Hypoxia | Normoxia | Hypoxia |
| 0h 0Gy | G2/M: | 25.04 ± 1.67 | 20.61 ± 0.72 | 26.27 ± 4.22 | 19.68 ± 0.80 | 22.59 ± 2.13 | 25.91 ± 4.08 | 26.67 ± 8.55 | 20.63 ± 0.67 |
|  | S: | 17.74 ± 1.52 | 11.23 ± 0.76 | 23.14 ± 0.70 | 13.95 ± 0.74 | 16.88 ± 2.52 | 12.29 ± 2.93 | 15.98 ± 2.82 | 11.46 ± 0.54 |
|  | G1: | 53.52 ± 1.50 | 64.64 ± 1.00 | 46.35 ± 10.26 | 65.00 ± 0.85 | 55.79 ± 1.16 | 59.59 ± 3.70 | 52.25 ± 4.55 | 63.60 ± 1.57 |
|  | Apoptosis: | 3.69 ± 0.61 | 3.52 ± 0.63 | 4.18 ± 0.68 | 1.37 ± 0.26 | 4.72 ± 1.63 | 2.20 ± 0.87 | 5.09 ± 1.89 | 4.30 ± 2.15 |
| 1h 10Gy | G2/M: | 23.86 ± 2.28 | 22.74 ± 1.48 | 25.19 ± 0.72 | 29.32 ± 0.73 | 18.69 ± 2.13 | 28.97 ± 0.49 | 29.24 ± 0.05 | 28.08 ± 0.86 |
|  | S: | 12.66 ± 1.55 | 10.95 ± 1.68 | 11.75 ± 0.43 | 16.65 ± 1.43 | 15.81 ± 2.42 | 9.38 ± 0.15 | 14.28 ± 0.01 | 15.86 ± 0.44 |
|  | G1: | 58.65 ± 0.03 | 63.77 ± 2.12 | 57.48 ± 0.54 | 49.84 ± 0.18 | 61.97 ± 2.01 | 60.36 ± 0.29 | 52.16 ± 0.03 | 51.40 ± 2.82 |
|  | Apoptosis: | 4.82 ± 0.70 | 2.52 ± 0.77 | 5.57 ± 0.26 | 4.19 ± 2.21 | 3.53 ± 1.28 | 1.29 ± 0.05 | 4.31 ± 0.02 | 4.66 ± 2.65 |
| 2h 10Gy | G2/M: | 18.46 ± 1.29 | 23.49 ± 3.73 | 27.20 ± 0.65 | 29.91 ± 1.31 | 19.36 ± 1.10 | 21.08 ± 0.05 | 32.17 ± 0.62 | 32.05 ± 1.35 |
|  | S: | 16.46 ± 4.82 | 21.42 ± 3.50 | 16.90 ± 0.33 | 16.69 ± 1.10 | 15.65 ± 0.30 | 15.44 ± 0.02 | 12.09 ± 1.18 | 10.98 ± 1.79 |
|  | G1: | 60.76 ± 6.11 | 52.05 ± 0.02 | 51.82 ± 0.83 | 51.42 ± 1.02 | 62.12 ± 0.18 | 61.10 ± 0.06 | 49.04 ± 0.96 | 52.52 ± 1.56 |
|  | Apoptosis: | 4.32 ± 0.58 | 3.04 ± 0.25 | 4.09 ± 0.51 | 1.97 ± 0.26 | 2.87 ± 1.22 | 2.37 ± 0.03 | 6.69 ± 0.85 | 4.45 ± 1.61 |
| 6h 10Gy | G2/M: | 26.10 ± 0.49 | 36.32 ± 1.63 | 19.87 ± 0.03 | 30.96 ± 6.66 | 25.66 ± 1.62 | 28.44 ± 3.03 | 32.93 ± 11.30 | 29.39 ± 1.19 |
|  | S: | 10.83 ± 1.79 | 15.36 ± 2.64 | 19.10 ± 0.05 | 20.04 ± 3.29 | 15.77 ± 2.13 | 18.16 ± 4.79 | 13.45 ± 0.83 | 15.69 ± 1.12 |
|  | G1: | 60.09 ± 1.73 | 44.32 ± 1.92 | 58.52 ± 0.02 | 47.52 ± 3.15 | 53.41 ± 1.97 | 51.64 ± 7.47 | 49.63 ± 9.44 | 52.52 ± 1.30 |
|  | Apoptosis: | 2.99 ± 0.10 | 3.98 ± 1.15 | 2.50 ± 0.00 | 1.48 ± 0.56 | 5.15 ± 1.79 | 1.75 ± 0.34 | 3.98 ± 1.02 | 2.39 ± 1.47 |
| 24h 0Gy | G2/M: | 19.72 ± 2.59 | 17.57 ± 1.05 | 19.65 ± 1.29 | 18.84 ± 0.33 | 25.46 ± 2.61 | 31.34 ± 0.45 | 30.95 ± 0.00 | 19.00 ± 0.20 |
|  | S: | 11.31 ± 2.14 | 13.89 ± 0.75 | 16.53 ± 1.01 | 11.21 ± 0.12 | 9.82 ± 2.65 | 8.55 ± 0.30 | 8.99 ± 0.01 | 8.08 ± 2.1 |
|  | G1: | 65.88 ± 3.54 | 64.29 ± 5.70 | 59.63 ± 0.64 | 57.04 ± 3.19 | 60.86 ± 3.30 | 58.32 ± 1.18 | 55.02 ± 0.01 | 67.46 ± 2.44 |
|  | Apoptosis: | 3.08 ± 0.63 | 4.24 ± 0.11 | 4.18 ± 1.96 | 12.91 ± 1.26 | 3.87 ± 1.54 | 1.78 ± 0.40 | 5.03 ± 0.01 | 5.46 ± 0.97 |
| 24h 10Gy | G2/M: | 50.00 ± 7.19 | 52.28 ± 1.25 | 56.00 ± 2.87 | 51.95 ± 2.33 | 68.9 ± 0.76 | 54.45 ± 4.79 | 52.40 ± 0.00 | 44.94 ± 0.03 |
|  | S: | 16.00 ± 2.23 | 6.63 ± 0.47 | 10.56 ± 2.24 | 12.09 ± 1.98 | 14.00 ± 0.01 | 21.43 ± 2.1 | 7.09 ± 1.74 | 16.21 ± 0.00 |
|  | G1: | 25.00 ± 3.67 | 36.73 ± 1.27 | 27.03 ± 0.82 | 28.14 ± 0.36 | 12.08 ± 0.05 | 21.68 ± 6.04 | 30.89 ± 0.00 | 35.31 ± 0.01 |
|  | Apoptosis: | 9.00 ± 2.57 | 4.35 ± 0.46 | 6.41 ± 3.01 | 7.82 ± 3.96 | 5.02 ± 0.02 | 2.43 ± 0.56 | 9.61 ± 0.94 | 3.53 ± 0.00 |
